# Plant species introduced by foreigners according to folk tradition in Norway and some other European countries: xenophobic tales or not?

**DOI:** 10.1186/s13002-015-0056-9

**Published:** 2015-10-05

**Authors:** Torbjørn Alm

**Affiliations:** Tromsø museum, University of Tromsø, PO Box 6050, Langnes N-9037 Tromsø, Norway

**Keywords:** Plant introductions, Anthropochores, Polemochores

## Abstract

**Background:**

In their quest to understand and interpret nature, people have frequently sought religious or divine origins for plant species and their characteristics. Less often, historical events or persons are involved. This study comprises eleven cases of the latter kind, all claiming that plant species have been introduced by foreigners or at least from foreign lands.

**Methods:**

Based on literature data and a few cases recorded during my own ethnobotanical field work, eleven European examples of pseudo-historical plant origins are presented here, including *Cakile maritima, Cicuta virosa, Lathyrus japonicus, Leymus arenarius, Primula vulgaris*, and *Scopolia carniolica* in Norway, *Heracleum mantegazzianum* and/or *H. persicum* in Denmark, *Phoenix dactylifera *and *P. theophrastii *in Greece, and *Jacobaea vulgaris *in Scotland.

**Results:**

The only common trait in these stories is that foreigner or at least foreign lands are claimed as sources of the plant species. In most cases, the “historical” explanations given in folk tradition are demonstrably at odds with reality. In those cases that involve poisonous or potentially harmful species (*Cicuta virosa, Heracleum mantegazzianum *and/or *H. persicum, Jacobaea vulgaris*), or the “useless” *Phoenix theophrastii*, with its inedible fruits, the stories may be interpreted as xenophobic, blaming foreigners for introducing dangerous or worthless species. The remaining examples merely suggest a search for exotic and seemingly rational, if erroneous, origins for plant species and stands that people considered strange and unusual.

**Conclusion:**

The spreading vectors assumed in folk tradition are correct and well documented, e.g. ship cargos (including goods and packing materials), which are responsible for introducing ballast plants and other anthropochores, and wartime activities, introducing a broad range of species (polemochores). They do not, however, apply to the species included in this study, which are either indigenous plants or introduced ornamentals. The foreigners appearing in the folk tales serve mostly as suitably exotic explanations for what is perceived “alien” plants.

## Introduction

From the earliest times, man has sought to interpret and find a meaning in his surroundings – not least the gifts of nature. Early interpretations have survived in the form of myths and mythology, explaining e.g. the presence or characteristics of some plant species as the gift or creation of the gods. Ancient mythologies provide fine examples. According to Greek mythology, the olive *Olea europaea* L. was a gift of the goddess Athena, presented to the city of Athens in a competition to show which god could provide the most useful gift [1].

The gods are not alone in being credited – or blamed – for the introduction of plant species. Folk tradition may provide seemingly rational explanations for the presence of certain plant species, claiming that they have been introduced e.g. by foreigners, or from foreign countries. This paper will examine eleven examples of such supposed historical plant origins, in Norway and some other European countries. Some stories are short, providing only snippets of information, and others are more elaborate, providing a wealth of – often contradicting – details.

## Material and methods

This study is based primarily on literature data. The Norwegian examples have been extracted from my own database of more than 7000 publications comprising information on plant names and plant uses in Norway, supplemented by similar stories recorded during my own ethnobotanical field work. I have included all Norwegian examples known to me: cowbane *Cicuta virosa* L. (Apiaceae), sea rocket *Cakile maritima* Scop. (Brassicaceae), sea pea *Lathyrus japonicus* Willd (Fabaceae), lyme grass *Leymus arenarius* (L.) Hochst. (Poaceae), primrose *Primula vulgaris* Huds. (Primulaceae), and henbane bell *Scopolia carniolica* Jacq. (Solanaceae). Of these, the *Cicuta* legend is noted in several 19th century publications, e.g. some travel accounts, but seems to have failed the attention of botanists, with the sole exception of Fredrik Christian Schübeler in 1888 [2] and a few dismissive lines in the works of Amund Helland in 1913 [3] and Torstein Lagerberg et al. in 1956 [4]. The legends related to *Leymus arenarius, Cakile maritima,* and *Lathyrus japonicus* derive from my own material.

It is worth noting that two major contributors to Norwegian ethnobotany both knew and dismissed such stories. The story related to *Primula vulgaris* is included in the vast material collected in the mid-20th century by Ove Arbo Høeg, but was not included in his published work [5], and Rolf Nordhagen, as editor of the Norwegian version of [4], was aware of the *Cicuta* legend, roundly dismissing it as “just an invention” ([4]: 44–45). Thus, similar stories may well have been neglected or overlooked, at least by botanists.

Four examples from other European countries are included here: Cretan date palm *Phoenix theophrasti* Greuter and date palm *P. dactylifera* L. (Araceae) in Greece (Crete), giant hogweeds *Heracleum* spp. (Apiaceae) in Denmark, and common ragwort *Jacobaea vulgaris* Gaertn. (syn. *Senecio jacobaea* L.) (Asteraceae) in Scotland. All of these have been extracted from published sources, in particular travel accounts – which often contain comments on “facts” that arouse the curiosity of both visitors and the local population. The selection makes no claim of completeness in terms of what may or may not be hiding in each country’s tradition. Due to Denmark and Norway’s joint history of almost 400 years as a twin kingdom (1536 to 1814), ruled from Copenhagen, Danish and Norwegian plant traditions are closely related, and knowledge of one will often elucidate the other. Vagn J. Brøndegaard’s compilation of Danish ethnobotany [6] provides an easy access to much Danish material, though my choice of examples may have been influenced by a long-standing interest in the invasive *Heracleum* species found in NW Europe [7, 8], presently been studied using DNA techniques to unravel their identity, origin and migrations routes [9]. The Greek examples form a striking parallel known to me from my 1998–1999 residence at the University of Crete (and later visits). I have added a British story simply because it fits my theme, and is easily accessible through Grigson’s treasure trove of British plant lore [10]. Similar stories are probably being told elsewhere, and may well be worth the attention of fellow ethnobotanists.

Citations from non-English literature are translated here; original language is indicated if it is not Norwegian.

## Results – or the plant stories

### *Cicuta virosa* and a band of Scottish mercenaries (Norway)

*Cicuta virosa* L. has a rather restricted distribution in Norway [11], extending into the country from the east (Sweden and Finland) in interior SE Norway and the far north (interior Finnmark). Due to its toxicity, it is well known to the inhabitants of the southern part of its Norwegian distribution area [12]. An especially large stand was found at Selsmyrene in the Gudbrandsdalen valley until the mid-20th century, when the swamps were drained and cultivated. The vernacular name used in Norwegian floras, *selsnepe* (“Sel’s turnip”), is a reminder of this. The name may be traced back to the late 17th century, but is probably much older. It is mentioned by Jonas Ramus in 1735 ([13]: 260) and Erik Pontoppidan in 1752 ([14]: 200ff). The latter cited a long letter from a local clergyman on the plant and its medicinal uses, both externally to relieve pain, and internally, in small doses, to cure a man affected by some kind of ill-defined, violent attacks. Pontoppidan also noted that the name was mentioned in a letter dated 1675, written by one of his relatives. Another early record is found in a letter from bishop and botanist Johan Ernst Gunnerus in Trondheim to Linnaeus, dated May 19, 1764, commenting on vernacular names, including *selsnepe* and *sprengrot* ([15]: 42); both were included in the brief account given in his *Flora norvegica* ([16]: 27).

Two mid-18th century topographical descriptions of the Gudbrandsdalen area have recently been published [17]. Both contain comments on *Cicuta.* The account of Christen Pram is dated 1743, and includes a separate paragraph entitled “Description of the so-called *Zællsnæper*”, commenting on livestock poisoning and the plant’s use as a cure for rheumatism ([17]: 303). The second manuscript, by Nils Hauritz, is undated, but probably also written in 1743. It contains similar information on toxicity and use ([17]: 332)*.* N.C. Lassen, who travelled through Gudbrandsdalen in 1777, mentions *Zels næper* and *Spræng-Rod* as local names ([18]: 18). The former was also noted (as *Selsnæpa*) in an early survey of Norwegian vernacular plant names by Ivar Aasen in 1860 ([19]: 16), and has since (as *selsnepe*) become the official Norwegian name of the species.

According to a local legend, *Cicuta virosa* was introduced to Norway and the site at Selsmyrene by Scottish mercenaries. They were attempting to march across the country in 1612, to aid the king of Sweden during the Kalmar war ([2]: 213–214). Memories of the Scottish mercenaries and their fate loom large in local legend. A band of 300 to 400 Scots were ambushed at Kringen in Sel on August 26, 1612, where the peasants rained logs and weapons on them. All but 18 Scots are supposed to have perished. The connection with *Cicuta* is first mentioned in Hans Peter Schnitler Krag’s 1838 collection of folklore related to the Scottish expedition:

“The origin of the plant called cow-bane or water hemlock (*Cicuta virosa* s. *aquatica*), which is very poisonous, and which grows in great quantities in a marsh at Nordre (North) Sel, dates, according to tradition, from the time of the Scots. It is said the Scots sowed that herb; but that this has only been attributed to them out of hatred need scarcely be added.” ([20]: 54, English translation from [21]: 123).

The legend was also known outside Gudbrandsdalen. In Romsdal, western Norway, where the Scots made their landfall before marching inland, Andreas Faye recorded a more detailed story:

According to a legend, Sinklar [Sinclair] brought with him to Norway a witch, who told his fortune every day, but on the eve of the battle at Kringlen (August 26, 1612), she could not make a prediction. Embittered by the Scots’ defeat, she sowed *Selsnæpen* [*Cicuta*] at Sel.“ (…) ”Another version of the legend has mixed this with Lady Sinklar [Sinclair].” ([22]: 192).

The legend was also recorded by Swedish author Viktor Rydberg, during a holiday visit to Norway in 1858:

“Another memory left by Sinclair’s men, is an addition to the flora of Norway, which is supposed not to occur anywhere else in Norway than in the area which the Scots marched through. This plant (…) is ill reputed for its toxicity. Livestock, which has consumed it, die mercilessly. Its head-quarters are found in a swamp at Laurgård. Attempts to eradicate it has not succeeded. It is as impossible to eradicate as ancestral sin, by which it, if I remember correctly, has been compared by doctor Wieselgren. It is a main ingredient in the despicable pharmacope of the gypsies, and its poisonous properties is enough to explain why, with such security, travelling gypsy bands can predict the death of the peasants’ cattle” (cited from [23]: 105; originally published as a series of newspaper notes).

Ten years later, in 1868, the Norwegian scholar Knud Knudsen was told a similar story. He obviously found the tale somewhat unlikely, as shown by a comment in his travel account:

“It was probably at this time, I gave a boy 4 sk[illing] to go into the tarn just by and collect a *sælsnæpe*, so that I could see, what this notorious (“infamous”) turnip looked like (cicuta virosa). Legend blaims the Scots of 1612 for having introduced this poisonous thing, but it was probably there before, and not just only in Gudbrandsdalen. The root looks like a turnip, and Laurgård lies close to the northern end of Sælsmyrene. This is probably also the origin of its vernacular name in Gudbrandsdalen. Otherwise, they told me that the cattle would search for it, but die if they consume it, unless an antidote is available.” (cited from [24]: 418)

In his collection of folklore related to the Scottish expedition, published in 1899, Andreas Austlid adds little new, but at least confirms that the legend was still being told:

“At Sells-vollom there grows a root, which is called *sellsnæpa*; she is extremely poisonous. When a sheep eats some of it, it will swell, and die at once. The people at Sell says, that the Scots sowed this turnip, when they left.” ([25]: 34)

Contrary to the legend, which ascribed leadership of the Scottish expedition of 1612 to Sinclair, a captain who was killed at Kringen, the leader of the Scots was a colonel, Alexander Ramsay, who survived and was brought as a prisoner to Copenhagen [21]. The plant part of the story is even less accurate. *Cicuta virosa*, although uncommon in Norway [11], is certainly not restricted to the area crossed by the Scots, and there is no reason to suggest they had anything to do with its introduction in Norway. Macrofossil remains reveal its presence at much earlier dates [26–28]. The species does occur in the British Isles, including Scotland, but is rather infrequent there [29]. Though no records are available, it can safely be assumed that *Cicuta virosa* was present at Sel long before the Scots’ ill-fated expedition.

It is worth noting than none of the 18th century sources quoted here contains any reference to the legend of a Scottish origin, even though they contain much information both on the *Cicuta* stands at Sel and the plant’s use in folk medicine. Pontoppidan’s 1750’s natural history of Norway [14] devoted four pages to the plant, and the account given in the printed version of Pram’s manuscript [17] runs into a full page of text. Thus, the legend was probably unknown to the 18th century authors, and we may assume that the legend was formed at some time during the next 80 years or so, and was fully established at the time of Krag and Faye, i.e. in 1838 to 1844 [20, 22]. Modern studies of the Scottish expedition and its fate leave little doubt that folk tradition has been busy constructing a legendary version of the events, altering and adding details as it saw fit [30, 31] – and the *Cicuta* story is but one addition.

### *Leymus arenarius* and a Dutch wreck (Norway)

Lyme grass is the main sand-binder on sandy shores along the northern coast of Norway, and may form vast stands at the larger sand-fields. It may also extend far inland (up to 200 km) along some of the major rivers. The species is more or less ubiquitous at the outer coast ([32]: 1118), which has not prevented people from, at least locally, inventing legends as to its origin.

**Fig. 1:**
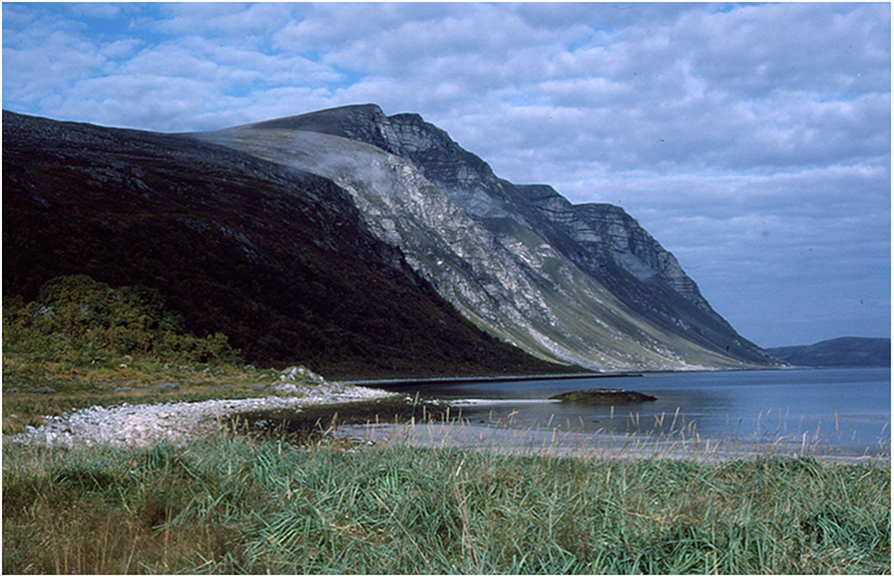
According to local lore at Vannøya in Karlsøy, Troms, northern Norway, the large stands of *Leymus arenarius* at the sand dunes at Skipsfjorden represent a kind of grain which sprouted from the cargo of a wrecked Dutch ship – ignoring the fact that the species is indigenous and widely distributed along the Norwegian coast. Photograph: Torbjørn Alm September 6, 2001

During field work at the island of Vanna at the NW coast of Troms, northern Norway, I visited the interior part of Skipsfjorden in September 2001. At the fjord head, *Leymus arenarius* forms a large stand at a sand dune area (Fig. 1). Three locals interviewed here all agreed on its origin: it was some kind of exotic grain originally introduced by a Dutch ship, wrecked off the coast, although the oldest of them would not exclude an alternative, Russian origin, and also found the story slightly suspect. The ship’s cargo of grain had supposedly drifted ashore, and given rise to the *Leymus* stands (EBATA 2001:9).

Again, the story may seem plausible to the locals, but does not stand up to scrutiny. Firstly, of course, *Leymus* is frequent elsewhere in the area, both at Vanna and adjacent islands [33, 34], and could presumably be found growing close to the homes of all three informants. This, however, would probably not deter them from claiming a Dutch origin; the cargo of grain could of course have drifted ashore over a larger area.

During the 16th and 17th century, Dutch ships often plied the waters off Norway, but mostly on whale hunting expeditions; remnants of Dutch whaling stations are found in several outer-coast stations in northern Norway (and in the Arctic archipelago of Svalbard) [35]. The likelihood of such ships carrying grain is much less. Although most grain consumed in the northernmost part of Norway at the time was imported, it was brought into the country by the German merchants of Bergen, traded for fish, and transported northwards with Norwegian vessels. The grain itself was mostly rye (*Secale cereale* L.) and some barley (*Hordeum vulgare* L.), and certainly not *Leymus arenarius*, which was only used locally as a grain substitute [36].

### *Cakile maritima* and a Spanish wreck (Norway)

A legend somewhat similar to that of *Leymus arenarius* is related to another coastal sand dune area, further south in Troms:

“Long ago a ship was wrecked at Sandsvika. The whole crew perished, except one. It turned out to be a Spaniard. He settled at Sandsvika. (…) As part of the story of this shipwreck, it should be added that the ship carried some barrels with seeds. These seeds drifted ashore and sprouted on the sandy slopes. From far back in time, one has heard that there were plants growing in Sandvika that are found nowhere else at the outer coast.” ([37]: 66–67).

Sandsvika is situated at the outer side of the major island of Senja. This rather inaccessible bay formerly held several farms, but is now desolate. The story told is rather similar to the one recorded at Vanna. Once upon a time, a Spanish ship was wrecked outside the bay. A single Spanish seaman survived, and settled in the area. Thus, some people here supposedly still show “Spanish” traits, and the local habit of taking a mid-day siesta is ascribed to him. Furthermore, the ship carried several barrels of seed. The legend, or a part of it, is also mentioned in an earlier account of the area, but in more general terms [38]. According to this version, people from southern Europe had settled in Sandsvika, possibly after being ship-wrecked. No mention is made of any botanical introductions.

Intrigued by this, I interviewed an old woman who had grown up in Sandsvika. She confirmed the legend, which she had heard from her father and grandfather. When asked about the plants, she described them as growing just above sea-level, on a substrate of kelp; they were “sea-plants” with blue or violet flowers (EBATA 2005:12), and a sweet, clover-like scent (EBATA 2005:17). A man from the same area also knew the legend, but placed the wreck in another bay, Testevika. He had also heard that the ship was Spanish, and that local rumours would have it that some mounds seen in the area are graves (EBATA 2005:20).

**Fig. 2:**
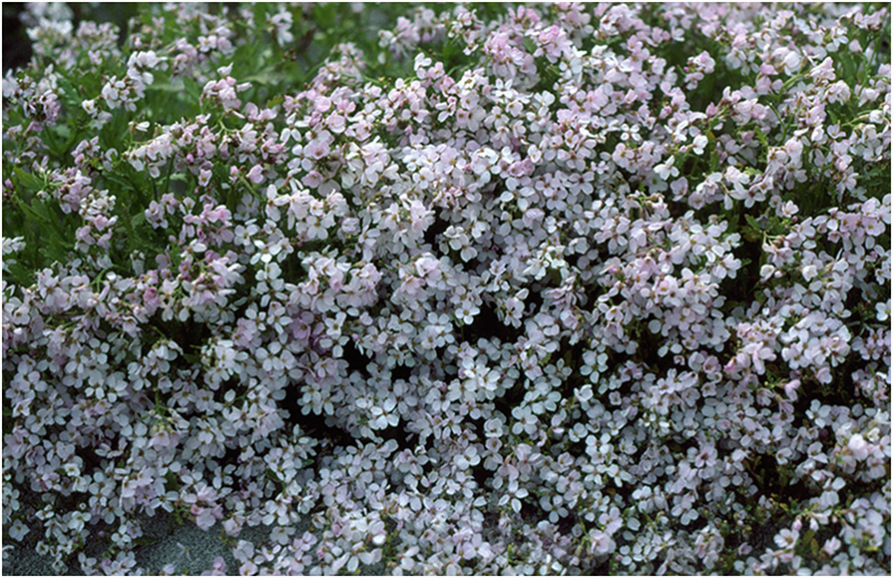
At Senja in Troms, northern Norway, people believe that a “strange” plant found in the Sandvika bay derive from a wrecked Spanish ship. In all probability, the tradition refers to *Cakile maritima*, which occurs abundantly here – and is in fact widespread along the coast. Photograph: Torbjørn Alm

The plant description given is hardly satisfactory in botanical terms. However, for anyone who knows the coastal sand dunes of North Norway and their flora, only two plant species stand out as likely candidates: *Cakile maritima* (Fig. 2) and *Lathyrus japonicus*. Both are showy, and thus likely to be noticed, and although widely distributed, they are certainly not found everywhere. In this particular case, the plant in question may be identified as *Cakile maritima*, which fits the description given (colour, scent), and is present at the site. Fjelland and others carried out a botanical investigation of Sandsvika, and reported unusually large stands of *C. maritima* ssp. *arctica* ([39]: 174), whereas *Lathyrus japonicus* was absent, and is generally rare at this part of the coast ([33]: 279–280]). Thus, the “exotic” species found here is a member of the indigenous flora, just as *Leymus arenarius* at Vanna. However, really large stands are confined to coastal sands, where their sweet scent may fill the air on hot summer days. As for other annuals, the stands may vary strongly in size from year to year. This is perhaps sufficient to explain why people considered it an exotic introduction, in need of a “historical” explanation.

**Fig. 3:**
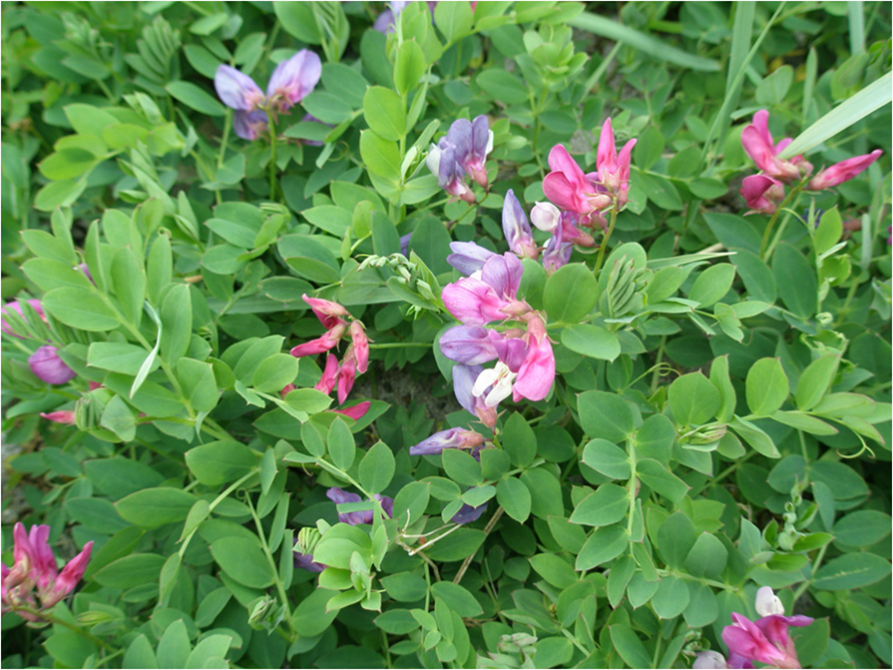
With its large, colourful and conspicuous flowers, *Lathyrus japonicus* may be the “strange” plant growing at Bøle in Hasvik, Finnmark, allegedly brought there by former whale-hunting expeditions, as seeds in their hay-stuffed matresses. Photograph: Torbjørn Alm

A somewhat similar story is told at the major Sørøya island of western Finnmark. According to local lore, “strange plants” occur at the remote outer-coast site of Bøle, in this case allegedly brought there with hay-stuffed matresses used by former whale hunters (EBATA 2005:56, 2005:57). In the version I was told, the visitors supposedly came from Vestfold in SE Norway, in which case the whaling would date no further back then the late 19th or early 20th century. It seems likely, though, that the story is older, and may earlier have claimed a Dutch origin, in accordance with the many Dutch whale hunting stations found along the northernmost coast of Norway – and the preference for exotic, foreign sources in other, similar tales. I have not visited the site, but a likely species to catch people’s attention here is *Lathyrus japonicus* (Fig. 3)*,* which is known to occur at Bøle ([40]: 362)]. Although not a rare species in Finnmark, its distribution is patchy [41], with about a hundred known stations distributed over a coast-line 6844 km. Thus, finding it may come as a surprise, not least due to its large and colourful flowers. Perhaps two species are involved, for some locals claim that there are two such “strange” plants at Bøle (EBATA 2005:56).

### Two further Norwegian examples

In addition to the examples cited above, Norwegian plant lore includes a couple of less elaborate examples of plants that, according to folk tradition, have been introduced by strangers or foreigners. *Scopolia carniolica* is an introduced species in Norway, cultivated as an ornamental and sometimes escaping from cultivation, as noted at scattered stations in southern Norway [32]. At least one such occurrence has received attention in local folklore. According to people at Oppdal in Sør-Trøndelag, the species, locally called *isblomst* (“ice flower”) and *giftblomst* (“poison flower”) had been brought there by a Russian soldier [42]. No further details are given, e.g. as to when it happened, or indeed why. Russian soldiers are not frequent visitors in any part of Norway, except as German prisoners of war during World War II. Such a mode of introduction is of course possible, but it is more likely that the plant derives from stands cultivated in gardens, just as elsewhere in southern Norway.

In the Mandal area of southernmost Norway, folk tradition claims that *Primula vulgaris* Huds. had been introduced from the Netherlands:

“*Liblomsten,* as we call it, has received its name from Skogsfjordliane. It is a Dutch flower. In the old days, there was substantial traffic of Dutch ships in Skogsfjorden. They came with ballast, and probably discharged the ballast somewhere at the head of the fjord. The ballast must have contained seeds, or roots of this flower. It has started to sprout, and over time, the plant has spread all over Skogsfjordliane and gained its name from them: *Liblomsten.*” [43]

A comment on this newspaper note is found in the vast ethnobotanical collection of the Norwegian botanist Ove Arbo Høeg (NFS O.A. Høg 194). According to it, the note was written by H. Isaachsen, a local agronomist. Høeg’s informant was somewhat skeptical as to the story’s veracity, and Høeg did not find it worth including in his published compilation [5]. *Primula vulgaris* is certainly indigenous in this part of Norway, and occurs at scattered stations along the coast all the way north to Trøndelag in central Norway [44]. The same vernacular name is known from other areas, and probably refers to ecology rather than any particular toponym or locality.

### Giant hogweeds *Heracleum* spp. and a cargo of statues (Denmark)

Giant hogweeds were fashionable garden plants in the early 19th century, due to their large size and vigorous growth. At least two species have been introduced to Denmark: *H. mantegazzianum* Somm. & Levier, which has become a troublesome pest, and *H. persicum* Desf., which is rare [45]. As for many introduced species, a considerable time lag occurred before their invasive nature was revealed, transforming the species from prized ornamentals into pest plants, with *H. mantegazzianum* forming rapidly expanding, uncontrollable stands invading a variety of habitats.

Vagn J. Brøndegaard noted an interesting legend explaining the introduction of a giant hogweed, assumed to be *H. mantegazzianum,* to Denmark ([6]: III, 307). According to Danish lore, the species was introduced in the 1830’s, when large quantities of “seeds” (i.e., fruits) were used to protect one or more cargos of sculptures sent home to Denmark by Danish artists. There are in fact two such stories; one attributing the introduction to an 1835 shipment containing sculptures by Herman W. Bissen sent home from Italy, and a similar story related to the works of Denmark’s famous sculptor Bertel Thorvaldsen (1770–1844). They were brought home about the time of his return from Italy in 1838; he had spent more than forty years working there.

In a recent review of the history and distribution of *H. mantegazzianum* in Denmark, Bruun and others [45] made a brief comment on the Thorvaldsen legend, rejecting it as inplausible. Seeds have certainly sometimes been used to protect valuable objects, e.g. exotic drift seeds in Norway ([46]: 253), but the quantity of *H. mantegazzianum* fruits needed to protect Thorvaldsen's numerous life-size or larger statues would have been vast. It would at least have required that *H. mantegazzianum* was growing abundantly in Italy at the time – which it was not [45], being neither native nor much cultivated. In fact, the species’ main connection with Italy is its name, given in honour of the Italian doctor Paolo Mantegazzi. If the statues were wrapped in plant material, hay or seaweeds are more likely alternatives, just as the glass factories of Venice traditonally used *Zostera marina* L. as packing material [47].

Thus, the Thorvaldsen legend is likely to be yet another example of a would-be historical explanation of a plant’s origin. This does not exclude the possibility that the cargo of statues did carry some seeds with them, but of other plants. They did. A British visitor to Denmark, Hugh Macmillan, devoted most of his brief 1873 travel account to commenting on Thorvaldsen, his museum and statues [48]. He also mentioned seeds and plants brought with them:

«When the statues which Thorvaldsen sent from Rome were unpacked in Copenhagen, several flowers sprang up very soon after in the neighbourhood formerly unknown. It seems that the sculptures were carefully wrapped round with bands of hay from the Campagna, containing the seeds of plants peculiar to Italy.» ([48]: 188–189)

Notably, no mention is given of *Heracleum mantegazzianum,* which is unlikely to have been among the plants deriving from Italy. It is certainly likely that giant hogweeds made their first appearance in Denmark in the 1830’s, or perhaps even earlier – they were popular in northern Europe at the time [8, 49]. In all probability, they were imported for garden use, and cultivated as ornamentals. Their identity is another matter. The first plants in Denmark may in fact have been *Heracleum persicum* Desf., rather than *H. mantegazzianum,* just as in Norway [8, 9].

### *Phoenix theophrasti* Greuter and *P. dactylifera* L. in Crete (Greece)

**Fig. 4:**
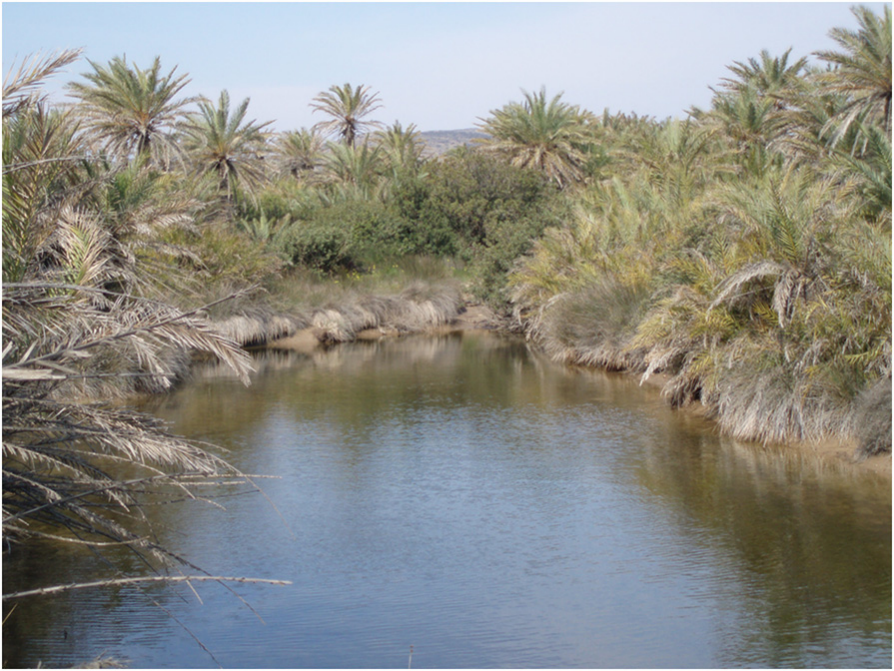
The palm grove at Vai, Crete (Greece), which according to legend sprouted from dates left here by Arab mercenaries. The folk tradition is slightly marred by the fact that these palms do not belong to the date palm *Phoenix dactylifera,* but a separate species (*Phoenix theophrasti*) with inedible fruits. Photograph: Torbjørn Alm, April 3, 2007

*Phoenix theophrasti* is a palm species closely related to the date palm *P. dactylifera*. It is endemic to the Eastern Mediterranean, with most of its known stations in Crete. It is also known from some other Aegean islands, and from mainland Turkey [50]. The most famous stand is undoubtedly the palm forest of Vai in eastern Crete (Fig. 4), comprising about 5000 palms within an area of 17 hectares [51]. It is a popular tourist attraction, drawing some 200,000 visitors annually. Smaller stands of *P. theophrasti* occur elsewhere in Crete [50, 52–55], which has not deterred people from claiming the palms at Vai to be unique, which is true only as far as the palm grove is concerned.

The palms at Vai provide a Greek example of explaining unexpected plant stands or occurrences in terms of foreign influence. According to legend, the palms at Vai were introduced by the Arabs, presumably during the period of Arab occupation of Crete, lasting from 824 to 961 A.D. [56]. The legend is mentioned (and discredited) in some guide-books to Crete, e.g. by Cameron ([57]: 274). More detailed reports are found in some travel accounts. The Danish author Ole Juul provides an example:

“Before going to the Touplou monastery, we make a diversion to the palm forest at Vai (…). A local fisherman told us that North African pirates once landed here to consume their provision of dates, the result of which was the wild palm forest, which surrounds the little jewel of a bay, with the bluest water and the whitest sand one could wish for. However, it is more realistic to suggest that an Egyptian ship with a cargo of dates has stranded off the coast on its way to the ancient Greek city of Itanos a few kilometres to the north.” ([58]: 172, translated from Danish).

Another Danish author, Bob Ramsing, provides a somewhat deviant account of local Cretan lore. With his Cretan wife, he was well positioned to gain access to local traditions. His version of the story is related both to the stands at Vai and at an unnamed village in the surroundings of Chania in Western Crete, starting at the latter:

“At the edge of the square in front of the [Agia] Marina church a vast palm is standing, and Petros says, that it derives from one of the date stones, which the soldiers of Napoleon spat out when they visited Crete and consumed the provision of dates they had brought from Egypt. Petros is the story-teller of the village, and my key to the Cretan world of legends. It is often questionable, if he keeps to the truth, but his store of stories is inexhaustible, and in this case there is at least a fragment of truth in what he says. The story of Napoleon’s soldiers and the date kernels is utterly suspect, for as far as I have been able to find out, Napoleon has never sent soldiers to Crete. Still, there is an element of truth to Petros’ story. In the 10th century, the infamous Arab mercenary Abu Hafs Omar had been driven out of both Egypt and Spain, and he then tried his luck in Crete, where at first he landed with his soldier in the island’s eastern corner at Vai. During their first time on the island, the Arabs lived on their provision of dates. According to legend, they sat at the shore and ate their dates, spitting the stones out on the sand. Today, there is a dense palm forest at Vai, which reminds you of the African coasts. The people of Abu Hafs Omars stayed in Crete for more than 100 years, and even if they remained at the coast, they were rapidly mixed with the population. And just as the palms at Vai spread to the rest of the island, there are now several villages with people of a typical Arab complexion.” ([59]: 38–39, translated from Danish).

Juul’s account [58] is interesting in providing both a record of local lore, and an attempt at a modern, rational explanation, even if both are equally at odds with reality. Being an endemic species with inedible fruits, the palms at Vai have nothing but a generic relationship with the date palm. In fact, the palms in Crete have been known since antiquity ([1]: 213), and are mentioned by Theophrastos, ‘the father of botany’ (see [60]: 141) – who was honoured by Greuter when naming the species [52]. Palms, possibly *P. theophrasti,* are also depicted in frescos in the Minoan palace at Knossos [61].

As noted by Ramsing, a “Napoleonic” origin of the palms in Crete, whether *P. theophrasti* or ordinary date palms *P. dactylifera,* is unlikely [59]. Napoleon’s ill-fated early 19th century naval expedition to Egypt had substantial political and archaeological ramifications, but hardly any influence in Crete. Ramsing’s belief in the “Arab” legend is hardly more than a tribute to the convincing story-telling of his Cretan source. At Vai, the locals still prefer the “Arab” story to the bleak botanical realities, as noted during my 2007 visit to the area.

### *Jacobaea vulgaris* Gaertn. and the Jacobite rebellion (Scotland)

This decorative, but poisonous plant is a troublesome weed in pastures. In Scotland, legend suggest that is was introduced by English troops after the Scots’ defeat at the battle of Culloden in 1742, during the Jacobite rebellion ([10]: 389, [62]: 122). The vernacular name used in Scotland, *Stinking Willie*, refers to William, the Duke of Cumberland, who was commander of the English troops, and merciless in his slaughter of the defeated Scots. The English on their part are supposed to have renamed the species *sweet William* in honour of the duke ([63]: 305).

According to folk tradition, the seeds of *Jacobaea vulgaris* were introduced with horse fodder brought by the English. Whether or not *J. vulgaris* was present in Scotland before the battle of Culloden (it probably was), the story does have a certain aura of credibility. Horse fodder is a well-known vector for introducing plant species.

## Discussion

I initially suspected that the kind of plant origin legends included here would have a xenophobic twist, attributing or blaming the introduction of unusual or harmful plants on foreigners, adversaries or perhaps even ethnic minorities. The examples included here do not bear this out, although in most of the eleven cases above, foreigners are blamed or claimed as the vector introducing “strange” plants to new areas. The story of giant hogweeds *Heracleum* spp. in Denmark do not explicitly blame foreigners, but does claim a foreign (Italian) origin. Otherwise, the species included in these tales do not have much in common, except that most are conspicuous in terms of habitus or size (*Heracleum mantegazzianum, H. persicum*, *Phoenix theophrasti, P. dactylifera*), or by forming large stands. Another common trait that may be significant applies to *Cicuta virosa, Scopolia carniolica,* and *Jacobaea vulgaris:* all three are poisonous. The first two are lethal if consumed in any quantity [64], whereas *Jacobaea vulgaris* may kill grazing cattle, horses and other livestock [65, 66]. Like *Heracleum mantegazzianum* and *H. persicum,* it may also cause dermatitis (skin sores and blistering) in humans [65–68]. Numerous *Lathyrus* species are poisonous ([69]: 555ff), and consumption may cause a serious neurotoxic disease called lathyrism [69, 70]. The peas of *L. japonicus* should probably not be eaten in any quantity (cf. [71]). The Cretans may also have had some reason to be disappointed with their native *Phoenix theophrasti,* with its inedible fruits. Thus, the legends of origin recorded for *Cicuta virosa, Lathyrus japonicus,* and *Scopolia carniolica* in Norway, giant hogweeds *Heracleum* spp. in Denmark, *Jacobaea vulgaris* in Scotland, and *Phoenix theophrasti* in Greece, may have some xenophobic tendency of blaming “foreigners” for introducing plant species that are perceived as harmful or at least disappointing.

*Leymus arenarius*, with its alleged Dutch origin, does not fit into such a pattern. On the contrary, its “seeds” (caryopsis) have to some extent served as a source of food in times of need [36], as recorded e.g. from Finnmark by W. Christy [72], though it was much more important in Iceland, also with a population of Norwegian origin [73–75]. It has been suggested that the “self-sown wheat” recorded during the Viking expeditions to North America about 1000 A.D. was *Leymus arenarius* ([75]: 30), or rather the closely related American *L. mollis* (Trin.) Pilg. – though this is unlikely, since finding a species looking much like the well-known *L. arenarius* would hardly cause any excitement and a record in the sagas. A number of other grass species has been suggested ([76]: 313–315). It is worth noting that *Leymus arenarius* is frequently infested with ergot (*Claviceps purpurea* Tulasne). In Iceland, where the seeds were still gathered in the early 20th century, people had realised that ergot was dangerous, and removed it when winnowing the harvest [74]. Failure to do so could certainly turn *Leymus* into a dangerous and potentially lethal crop, with the same disastrous consequences as consuming ergot-infected grain [77–79]. Thus, *L. arenarius* may have had some past reputation of being (potentially) poisononous, which would make it fit into the pattern outlined above. This leaves only the parallel story of *Cakile maritima,* claimed to be of similar origin, unexplained. The species is both harmless and edible.

A brief summary of the species discussed here is found in Table 1. Although the number of cases is limited, they derive from four different countries, and widely dispersed parts of Europe. Thus, it is probably more than a coincidence that the majority shows common traits: the species accounted for are often poisonous or potentially harmful, and their introduction is supposedly due to foreigners or foreign influence. *Cicuta virosa, Heracleum mantegazzianum, Scopolia carniolica,* and *Phoenix theophrasti* fits a common pattern of xenophobic, pseudo-historical plant origin myths – which, like many prejudices, do not stand up to closer scrutiny and the hard facts. The legend of *Jacobaea vulgaris* in Scotland fits the same pattern – though it might possibly be the rare exception where the time and mode of introduction of a noxious plant species is correctly reflected in legend.

**Table 1:** Summary of species included in this study

Taxon	English name	Country	Supposed origin in folk tradition	Phytogeography	Characteristics
*Cakile maritima*	Sea rocket	Norway	Foreign (Spanish), wrecked ship	Indigenous	Conspicuous in flower and in large stands
*Cicuta virosa*	Cowbane	Norway	Foreign (Scottish), mercenaries	Indigenous	Poisonous
*Heracleum mantegazzianum*	Giant hogweed	Denmark	Foreign (Italian), packing material	Introduced (Caucasus)	Harmful, conspicuous (size)
*Heracleum persicum*	Persian hogweed	Denmark	Foreign (Italian), packing material	Introduced (Iran)	Harmful, conspicuous (size)
*Jacobaea vulgaris*	Common ragwort	Scotland	Foreign (English), horse fodder	Probably indigenous	Poisonous
*Lathyrus japonicus*	Sea pea	Norway	Possibly foreign (Dutch), whalers	Indigenous	Conspicuous in flower; poisonous if peas are consumed in quantity
*Leymus arenarius*	Lyme grass	Norway	Foreign (Dutch), grain from wreck	Indigenous	Conspicuous in large stands, harmful if infested with ergot
*Phoenix dactylifera*	Date palm	Greece (Crete)	Foreign (Egyptian), soldiers	Introduced	Conspicuous in shape and size, cultivated ornamental
*Phoenix theophrasti*	Cretan date palm	Greece (Crete)	Foreign (Arab), pirates	Indigenous, endemic	Conspicuous in shape and size, inedible fruits
*Primula vulgaris*	Primrose	Norway	Foreign (Dutch), ballast	Indigenous	Conspicuous in flower
*Scopolia carniolica*	Henbane bell	Norway	Foregin (Russian), soldier	Introduced (southern Europe)	Poisonous, cultivated ornamental

There is obviously much sense in the folk notion of plants being introduced by man. Ships and their cargo are well-known spreading vectors, not least through discharged ballast – as (wrongly) suggested for *Primula vulgaris* at Mandal in southernmost Norway. This mode of introduction has been studied extensively in Norway [80–84], confirming it as the source of a substantial number of exotic, if generally short-lived species.

Folk tradition is also correct in claiming that war-time activities may introduce new species, as assumed for the alleged introduction of *Cicuta virosa* to Norway in 1612 by Scottish mercenaries in the service of the Swedish king, and *Jacobaea vulgaris* in Scotland – although at least the former story is certainly misleading. Vagn J. Brøndegaard noted that similar origins were claimed for several plant species in Denmark [85]. *Anchusa officinalis* L. was allegedly introduced to Denmark with invading Swedish troops in 1658–1659; the plant is thus called *svenskere* (“Swedes”) in parts of Denmark ([86]: 46); other Danish vernacular names would rather suggest an introduction with German soldiers: *husarblomst* (“hussar flower”), *tysk husar* (“German hussar“) and *tyskere* (“Germans”). A rather similar story is noted for *Echium vulgare* L. ([85]: 51). However, both species were locally frequent in Denmark in the mid-17th century, prior to their alleged introduction ([85]: 51). The shift from a supposed Swedish to German origin reflects historical changes, Germany supplanting Sweden as the main enemy. In similar fashion, Danish tradition claims that *Glebionis segetum* (L.) Fourr., formerly a troublesome weed, was a war-time introduction to Denmark, coupling it with cavalry from Brandenburg. Other versions blame its introduction on Swedish troops returning home from Germany through Schleswig and Denmark – and, for that matter, its introduction to Sweden on Danish troops ([87]: 1611). A somewhat later version claims that *G. segetum* came with the horse fodder used by Spanish troops stationed at Fyn in 1808 ([85]: 52–53). A similar story is used to explain the abundant local occurrence of *Papaver rhoeas* L.*,* allegedly sprouting in an area coloured red by the blood of the Spanish horses ([88]: 113–114). Many other plant species have received “military” vernacular names in Europe (for a review, see [85]), and are sometimes claimed as foreign introductions. Roy Vickery mentions a number of similar stories from Britain. The winter aconite *Eranthis hyemalis* (L.) Salisb. is supposedly restricted to sites drenched by the blood of Roman soldiers ([89]: 100), and several plant species allegedly grow where the blood of invading Danes was spilled. These include pasqueflower *Pulsatilla vulgaris* Miller*,* field eryngo *Eryngium campestre* L*.,* and dwarf elder *Sambucus ebulus* L. ([89]: 98–100). Travelling through southeast Sweden in 1741, Linnaeus noted that latter species was supposed to grow only at the castle of Kalmar, and nowhere else in the world, having sprouted from the blood of Swedes and Danes killed in a battle there ([90]: 35).

Plant introductions with foreign troops are in fact so common and well documented that the Finnish botanist Panu Mannerkorpi in 1944 coined a special term, *polemochores* (from Greek *polemos,* war, and *chorein,* to disperse) for them – in his case referring to eastern or ‘Russian’ plants introduced to Finland by Soviet troops during the 1939–40 winter war [91]. Similar introductions with German or Russian troops are well known from NE Norway [92], and they provide the only likely explanation for the rich flora of the so-called "wonderglades" of NW Russia [93]. Thus, although the historical veracity may be doubted for some of the supposed war-time introductions recorded in folk tradition, the mode of dispersal is certainly both possible and well documented.

Although some of the modes and routes of plant dispersal suggested by folk tradition may make sense, others do not. Roy Vickery provides a fine example from Britain, where the introduced everlasting pea *Lathyrus latifolius* L. is known by the locals of Northamptonshire as *Pharaoh’s peas.* The local presence of this colourful species, native to southern Europe, is explained as due to peas brought from Egypt, allegedly deriving for a royal tomb in a pyramid ([89]: 102) – again showing folk tradition’s preference for exotic origins and explanations.

In terms of phytogeography, folk tradition certainly takes a liberal stance, claiming that anything from common, native plants (e.g., *Leymus arenarius* in Norway) to local endemics (*Phoenix theophrasti* in Crete) are foreign introductions. It may, for that matter, also claim that imported plant species, often from very distant lands, are native, as is the case for some ornamentals and other cultivated plants, e.g. *Heracleum persicum* in North Norway [8]. Unlike the more or less poisonous and “useless” plants attributed to foreigners or foreign influence in the legends above, useful plants in general, no matter their origin, are eagerly accepted and internalised in folk tradition. As a consequence, the ethnobotany of Norway (and many other countries) abounds in traditions related to exotic taxa, e.g. to chili pepper *Capsicum annuum* L. [94], carob *Ceratonia siliqua* L. [95], camphor *Cinnamomum camphora* (L.) J. Presl. [96], coffee *Coffea arabia* L. [97–99], saffron *Crocus sativus* L. [100], turmeric *Curcuma longa* L. [101], asafoetida *Ferula assa-foetida* L. [102], laurel *Laurus nobilis* L. [103]*,* nutmeg *Myristica fragrans* Houtt. [104], myrtle *Myrtus communis* L. [105], black pepper *Piper nigrum* L. [106], potato *Solanum tuberosum* L. [107–110], ginger *Zingiber officinale* Roscoe [111], etc.

## Conclusion

Although foreigners and their activities have some support as spreading vectors for plants, they are hardly relevant to the stories outlined above. The general picture emerging is one of wonder – and an attempt at explaining plant occurrences or plants that people find unusual by equally unusual means, e.g., in terms of seeds drifting ashore from wrecked foreign ships (*Lathyrus japonicus* and *Leymus arenarius* in Norway), or food consumed by Arab invaders (*Phoenix theophrasti* in Crete). It may be concluded that in folk tradition, plant or plants stands that are considered unusual in some way, deserve equally unusual explanations. This certainly provides for better stories than the prosaic botanical realities – and good stories tend to be told and remembered. Olaf Olafsen, who collected folklore on the Scottish expedition through Norway, albeit without recording the legend related to *Cicuta virosa,* provides an apt comment:”Legends tend to relate everything extraordinary to those events or persons which, more than others, live in oral tradition” ([112]: 8).
